# “Feeling and Knowing. Making Minds Conscious”

**DOI:** 10.17505/jpor.2025.28333

**Published:** 2025-10-02

**Authors:** Lars-Gunnar Lundh

**Affiliations:** Department of Psychology, Lund University, Sweden

**Keywords:** Feeling, Consciousness, Sensing, Sentience, Homeostatic processes, Implicit intelligence, Damasio, Personhood

The neuroscientist Antonio Damasio, professor at the University of Southern California, is known for his contributions to research on the relation between feelings, consciousness, the body, and the brain. He has authored several books, of which the first one, *Descartes’ Error: Emotion, Reason and the Human Brain* from 1994, is the most well-known. After this book there has come five others: *The Feeling of What Happens: Body and Emotion in the Making of Consciousness* in 1999; *Looking for Spinoza: Joy, Sorrow, and the Feeling Brain* in 2003; *Self Comes to Mind: Constructing the Conscious Brain* in 2010; *The Strange Order of Things: Life, Feeling, and the Making of Cultures* in 2018; and *Feeling and Knowing: Making Minds Conscious* in 2021. It is the latter, his latest book, that is the subject of this review.

This is a concise book, with short chapters, rather different from Damasio’s previous books. One reason for keeping it short is expressed by the author right at the first page of the text, where he describes frustration in connection with the reception of his previous writings:

The frustration came from talking to many of my readers, over the years, and learning that some of the ideas that I wrote with such enthusiasm – and that I had been keenest to have readers discover and enjoy – were lost in the middle of long discussions and hardly noticed, let alone enjoyed. My private response, on such occasions had been a firm but always postponed decision: to write only about the ideas I most care for and leave behind the connective tissue and the scaffolding meant to frame them. (Damasio, 2021, p. 3).

This sets the stage for the present book: Here his ambition is to “do what good poets and sculptors do so well: chip away at the nonessential and then chip some more; practice the art of Haiku” (p. 3-4).

This condensation of contents means that some parts of the book might perhaps be difficult to fully grasp for someone who is unfamiliar with Damasio’s previous books. At the same time, the present book cannot be reduced simply to some kind of summary of his previous works. On the contrary, this little book clearly represents a further development of some aspects of Damasio’s thinking. As he points out in the introductory pages, “the effort of reconsidering and pairing down so much material”, in fact, helped to “confront facts that I had overlooked and develop new insights about not just consciousness but related processes” (p. 4). The book may therefore be valuable reading also for readers who are very familiar with Damasio’s previous works.

In this review, I will focus on three themes in Damasio’s book, which correspond to what he depicts as “three distinct and consecutive evolutionary stages”, labelled as *being*, *feeling*, and *knowing*, and which he also describes as corresponding to “separable anatomical and functional systems that coexist in each of us humans” (Damasio, 2021, p. 25). The first stage (or *layer*, as I guess it might also be called, as it is assumed to exist in all of us, from unicellular organisms to adult human beings), involves *sensing* and non-explicit intelligence in the form of *homeostatic* processes. The second stage or layer, which is found in organisms with nervous systems, is dominated by *feeling,* primarily in the form of subjective experiences of homeostatic bodily processes, and “minding” (in the form of images of the surrounding environment). Finally, the third stage or layer, labelled as *knowing*, involves the development of *consciousness*, defined in terms of an experience of ownership not only of one’s body but also of one’s feelings, images, thoughts, and memories. All three stages/layers are described by Damasio in ways that stimulate further reflection; in the following I will therefore also raise some general points of debate.

## Being, Sensing, and Homeostatic Processes

When the first living cells developed, around 3.8 billion years ago, this involved the development of what Damasio describes as *sensing*, and *homeostatic* processes that amount to a form of *non-explicit intelligence.* This is a kind of intelligence which is seen already in unicellular organisms such as bacteria and in plants, and that is expressed in the living organism’s tendency to maintain itself, and to stay alive. For this purpose, it relies on homeostatic processes that serve to keep various physiological parameters within the range most conducive to their optimal functioning.

Damasio is very clear that this kind of sensing is *not* perceiving, and that it is not a *mental* process (mental processes require nervous systems). At the same time, he sees this kind of sensing as a “most elementary variety of cognition” (Damasio, 2021, p. 13). Because it makes the organism respond in ways that serves “the continuation of their lives” (p. 14), he concludes that it involves *intelligence* – even if only a *non-explicit* form of intelligence. In other words, at this first stage of life we find *intelligence without minds* (“unminded” or “non-minded” intelligence).

This raises some interesting questions about the nature of intelligence. In this picture, intelligence developed before there were any mental processes. This kind of intelligence, in a certain sense, even seems to precede the development of *living organisms*, as seen in Damasio’s discussion of viruses. Unlike bacteria, viruses are not living organisms, but they still exemplify a simple form of intelligence, of the same kind that is found in bacteria:

Comparing viruses and bacteria is most informative. Viruses do not have energy metabolism, but bacteria do, viruses do not produce energy or waste, but bacteria do. Viruses cannot initiate movement… Viruses cannot reproduce on their own, but they can invade living organisms, hijack their life system, and multiply. In brief, they are not living but can become parasitic of the living and make a “pseudo” living while, in most instances, destroying the life that allows them to continue their ambiguous existence and promoting the manufacture of “their” nucleic acids. And on that point, in spite of their non-living status, we cannot deny viruses some fraction of the non-explicit variety of intelligence that animates all living organisms, beginning with bacteria. (Damasio, 2021, p. 20).

This illustrates not only how intelligence can develop independently of minds and mental processes, but also that some forms of intelligence obviously have developed outside of living organisms. Because this is an intelligence in the service of self-preservation, it also raises questions about the nature of the kind of “self” that strives for self-preservation. Maybe this kind of intelligence in the service of self-preservation can even be found at the level of genes, as seemingly implied by Dawkins’ (2016) concept of “the selfish gene”?

Another important aspect of this kind of non-explicit intelligence is that even adult human beings make heavy use of it, and that we benefit from its mechanisms “at all hours of the day” (Damasio, 2021, p. 3). In other words: The development of mental processes such as feeling, thinking, and consciousness, with the new forms of intelligence that they bring along, does in no way *replace* the old kinds of non-explicit intelligence, but rather builds new processes and structures on top of these old ones.

As to *sensing*, Damasio clearly takes the position that it involves nothing like feeling or consciousness. At the same time, he refers to research findings which show that both bacteria and plants are affected by anesthetics, which “suspend their sensing and place them in a literal hibernation” (Damasio, 2021, p. 168). In humans, anesthetics cause a temporary loss of sensation, which prevents pain during medical procedures. Now, if even bacteria and plants respond to anesthetics by losing *their* form of sensing, doesn’t this point to the possibility that *similar mechanisms* may be involved here? And if so, could this mean that the sensing that occurs in bacteria and plants is more similar to our own sensations than we might be tempted to admit?

According to Reber and Baluška’s (2021) Cellular Basis of Consciousness (CBC) model, all organisms are *sentient*, based on processes that take place in excitable membranes of their cells. Unfortunately, they equate sentience with *consciousness*, as also seen in the name of their model (“Cellular Basis of Consciousness”). Their model is therefore relatively easy to dismiss, at least with Damasio’s definition of consciousness (see below). More generally, this illustrates how discussions about the nature of consciousness all too often suffer from an insufficient differentiation between different kinds of phenomena. The questions involved here are complex, and further analysis may require more refined distinctions between different aspects of sentience, feeling, and consciousness. Godfrey-Smith (2024), for example, has argued that an evolutionary perspective

motivates a strongly gradualist view of consciousness; a simple distinction between conscious and nonconscious animals will probably be replaced with a view that admits differences of degree, perhaps on many dimensions (Godfrey-Smith, 2024, p. 1660).

Damasio contributes to such a differentiation by distinguishing clearly between sensing, feeling, and consciousness, and by tracing their evolutionary roots to three different stages of evolution. Of particular interest here is his assumption that consciousness (i.e., the most advanced stage) *builds* on the previous stages of sensing and feeling. The reason that anesthetics can impede consciousness, as he puts it, is that they “*target functions on which normal consciousness depends*” (Damasio, 2021, p. 168) – in other words, consciousness *depends on* sensing:

in the absence of sensing we cannot build up the operations that gradually enable plain minds, feelings, and self-reference, the ingredients that eventually permit conscious minds. (Damasio, 2021, p. 168-169).

This does *not*, however, mean that sensing represents some primitive form of consciousness. What it means is simply that consciousness depends on its biological *substrate*:

One bizarre consequence of the extraordinary success of the computational sciences is the idea that minds, including the human variety, would not depend on the substrate that supports them. (Damasio, 2021, p. 163).

Sensing is an essential part of *being*:

The “being” component is permanently present, even when it is not dominant... To say that our conscious minds would be substrate-independent would be to say that the edifice of “being” could be dispensed with… It would be to deny that the foundation of mental experiences is, to begin, *the experience/consciousness of a particular kind of organism in a particular state*. (Damasio, 2021, p. 164-165).

In other words: consciousness is the result of the development of *living organisms*, as part of evolution, which means that it relies essentially on biological processes at the cellular level.

## Feelings, Body, and Brain

Feelings, in Damasio’s picture, appeared with the evolution of multicellular organisms with nervous systems. The first multicellular organisms are assumed to have developed around 600-700 million years ago, whereas the first nervous cells developed around 500 million years ago. The nervous system made it possible for living beings to *feel their bodily interiors* – and, in particular, to feel the homeostatic processes in the body. Feelings, as described by Damasio (2021) are a kind of *hybrid* processes that are “at once mental and physical” (p. 191) and involve “back-and-forth interactions of the interoceptive nervous system with the actual viscera in our interior” (p. 175).

Damasio speaks about two categories of feelings: *Primordial* feelings and *emotional* feelings. Primordial feelings arose first, as spontaneous reflections of the homeostatic processes in the body; examples are feelings of hunger, thirst, pain and pleasure, tiredness and alertness. Emotional feelings came later, in connection with the development of emotions (such as anger, fear, and joy) in relation to the organism’s images of its interaction with the surroundings. Here I will focus only on Damasio’s discussion of primordial feelings.

Although Damasio connects the notion of primordial feelings primarily to subjective experiences of homeostatic processes, he also seems to include other basic feelings that are related to our very *being* (i.e., the first layer or stage of evolutionary development, as described above). This is summarized in the following way in one of his previous books, where he writes that primordial feelings

occur spontaneously and continuously whenever one is awake. They provide a direct experience of one’s own living body, wordless, unadorned, and connected to nothing but sheer existence. (Damasio, 2010, p. 21)

What made feelings possible, as Damasio describes it, was not the development of nervous systems in themselves, but their *interaction with bodily chemistry*. As he describes it,

Feeling probably began its evolutionary history as a timid conversation between the chemistry of life and the early version of a nervous system within one particular organism. In creatures far simpler than we are, the exchange would have generated feelings such as plain well-being and basic discomfort (Damasio, 2021, p. 71).

To me this suggests the possibility that feelings *emerged* (although this is a term that is not so often used by Damasio in the present book) as the result of the interaction of biochemical homeostatic processes with the bioelectric activity of neurons within living organisms – perhaps somewhat in analogy with the emergence of water as the result of the merger of oxygen with hydrogen? At least some formulations suggest that Damasio may have something similar in mind, as when he states that feeling “emerges… from a dynamic give-and-take between body chemistry and the bioelectrical activity of neurons” (p. 77).

One of Damasio’s main claims is that “*everything we feel corresponds to states of our interior*” (Damasio, 2021, p. 74). This does not mean, however, that feelings are to be understood according to the same model as we use for understanding the perception of the external world. *Intero*ception is very different from *extero*ception. Although the nervous system is essential both to our experience of the surrounding world and to the experience of our interiors, Damasio makes it clear that the nervous system’s interaction with the interior body is of a much more intimate nature than its interaction with the surrounding environment. As he formulated it in a previous work:

feeling is *not* a perception of the body in the conventional sense of the term. Here the duality of subject-object, of perceiver-perceived, breaks down. Relative to this part of the process, there is unity instead. *Feeling is the mental aspect of that unity.* (Damasio, 2018, p. 126).

If I understand Damasio correctly, this might also be illustrated by the apparent fact that to *feel* tired is to *be* tired; to *feel* hungry is to *be* hungry; to *feel* pain is to *be* in pain, etc. In other words: there is *no differentiation between the feeling and that which is felt*. In the perception of the external world, in contrast, there is always a differentiation between the perception and the object perceived – this means, for example, that misperceptions can occur, which can be corrected by a more detailed exploration of the external situation. Feelings are not like that – there is no such thing as feeling tired and finding out that one was wrong about it.

Damasio has used different formulations over the years in his attempts to catch the very special kind of relation between bodily feelings and the body as felt, and the underlying interaction between neurons and body chemistry. In one of his previous books, he spoke about the intimate association between the neurons conveying signals from the body’s interior to the brain as being in such an intimate association with the interior processes “that the signals conveyed would not merely be *about* the state of the flesh but literally *extensions* of the flesh” (Damasio, 2010, p. 257). In his latest book, he writes:

In practice, there is little distance between feelings and the things felt. Feelings are commingled with the things and events we feel thanks to the exceptional and intimate cross talk between body structures and nervous system. (Damasio, 2021, p. 71).

Damasio points to several possible physiological mechanisms underlying this “commingling”, in the form of peculiarities of the neurons that interact with our interiors (interoceptive neurons). One peculiarity is that most interoceptive neurons lack myelin insulation, which makes it possible for the molecules surrounding an axon to interact with it and alter its firing potential, so that the signaling of these neurons is “not really separate from the body that hosts them” (p. 93). Another peculiarity is that interoceptive neurons lack the barrier that typically separates neural processes from the bloodstream (the blood-brain barrier). As he summarizes it,

Lack of myelin insulation and lack of blood-brain barrier allow *signals from the body to interact with neural signals directly*. In no way can interoception be regarded as a plain perceptual representation of the body inside the nervous system. There is, rather, an extensive commingling of signals (Damasio, 2021, p. 94).

A related but more general observation concerns the unique relationship that obtains between the body and the nervous system, in the sense that

the former entirely contains the latter within its borders… As a consequence, body and nervous system can *interact directly and abundantly*. Nothing comparable holds for the relation between the world external to our organism and our nervous system. An astonishing consequence of this peculiar arrangement is that feelings are not conventional perceptions of the body but rather *hybrids*, at home in both body and brain. (Damasio, 2021, p. 7)

Perhaps most interesting of all, this involves *a two-way interaction* between the nervous system and bodily states, which means that our bodily states are influenced by our neural/mental processes:

Because the object and subject of our feeling-percepts exist within the same organism, *they can interact*. The central nervous system can modify the body state that gives rise to the particular feeling and, by doing so, modify what is felt. *This is an extraordinary setup that has no counterpart in the world of external perception*s. (Damasio, 2021, p. 89).

In other words, our neural/mental processes can modify not only our *bodily feelings* but also our *bodily states*. This makes interoception radically different from external perception.

Of course, we can modify our *experience* of the external world by shifting our attention between different parts of the surroundings, just as we can modify our *experience* of the body by shifting our attention between different parts of the body. If I understand Damasio’s reasoning correctly, however, it implies that we can modify *the physical state* of our body by means of mental processes in a way that we cannot modify the surroundings. To me, this seems to be an interesting point that may be theoretically relevant not only for the understanding of emotional and psychosomatic disorders but also for the development of treatments in this area.

Of particular interest here are body-focused practices, such as yoga, mindfulness meditation, Tai Chi and Quigong, where the participant attends specifically to their bodily states and processes. These practices have been the focus of increased research during the last decades, as to their effects on mental health and well-being. The theoretical understanding of the underlying mechanisms for these effects, however, is still largely missing. Maybe Damasio’s reasoning can open up for new research approaches to address such questions.

Consider, for example, the so-called body scan, as used in mindfulness meditation, where the individual is guided to move their attention systematically through all parts of the body, typically by starting from the toes and moving all the way up to the head. The instructions include “paying close attention to any sensations (or lack of sensations) in this area of your body” (Shapiro and Carlson 2017, p. 158), and to use the breath systematically by “breathing into” each body area in connection with attending to it. This way of scanning all regions of the body is to be done with an attitude of active interest, a gentle curiosity towards one’s sensations, and a friendly, caring attitude. This is an example of a body-focused *phenomenological practice* (defined as a practice that consists in deliberately modifying one’s attention and attitude to bodily experience; Lundh, 2020). An experimental study of the effects of such practices would require not only a study of subjectively experienced effects, but also of *physiologically* measurable effects. And a prediction that seems to follow from Damasio’s model, if I understand him correctly, is that we are likely to find physiological effects – because by modifying our attention and attitude to the body we also modify not only our bodily experience but also *aspects of our bodily states*. Exactly *how* phenomenological body-focused practices modify our bodily states is a question to be pursued in empirical research.

## Consciousness, Feelings, and Perspective

As Damasio pictures consciousness, it is essentially dependent on *feelings*, and feelings (as discussed in the previous section) are a hybrid between processes in the nervous system *and biochemical processes of the body* that the nervous system is part of. This means that consciousness cannot be based solely on the nervous system. Although the nervous system is a *critical contributor* to the development of minds and consciousness, Damasio makes it clear that

any theory that relies *exclusively* on the nervous system to account for minds and consciousness is also likely to fail. Unfortunately, this is the case with most theories today. (Damasio, 2021, p. 21).

This is an important critique of other theories in the field. It also entails a fundamental critique of the notion of “the hard problem of consciousness” as formulated by Chalmers when he posed the question “Why and how do physical processes in the brain give rise to conscious experience?” According to Damasio this formulation of the question is basically unsound:

Asking why should physical processes “in the brain” give rise to conscious experience is the wrong question. While the brain is an indispensable part of the generation of consciousness, nothing suggests that the brain generates consciousness alone. On the contrary, the non-neural tissues of the organism’s body proper contribute importantly to the creation of any conscious moment and must be a part of the problem’s solution. This happens most notably via the hybrid process of feeling, which we regard as a critical contributor to the making of conscious minds. (Damasio, 2021, p. 127-128)

Importantly, Damasio does not want to *reduce* consciousness to feelings. For one thing, conscious experience also involves *perspective:*

It is a state of *mind* imbued with two striking and related features: the mental contents it displays are *felt*, and those mental contents adopt one singular *perspective*. (Damasio, 2021, p. 120)

Furthermore, this perspective “is that of the particular organism within which the mind inheres” (p. 120), which points to the notion of a *subject* or *self,* to which this perspective *belongs*. This leads Damasio to the concept of “ownership” as a basic characteristic of the conscious mind:

What does it mean to say “I am conscious”? At the simplest level imaginable, it means to say that my mind, at the particular moment in which I describe myself as conscious, is in possession of knowledge that spontaneously identifies me as its proprietor. (Damasio, 2021, p. 128)

Among other things, this means that consciousness is not synonymous with mind. Consciousness is rather a particular state of mind which involves *self-reference as a defining, indispensable characteristic*:

If we were to remove the conscious component from our ongoing mental states, you and I would still have images flowing in our minds, but those images would be unconnected to us as singular individuals. (Damasio, 2021, p. 114)

This also means that he explicitly opposes those theories that speak about integration of information as the source of consciousness (e.g., Tononi, 2008). Increased integration of contents may well result in “an enlargement of the mental scope” (p. 157), but this is not sufficient to produce a conscious mind:

What does begin to engender consciousness is the enrichment of the mental flow with the sort of knowledge that points to the organism as the proprietor of the mind. What begins to make my mental contents conscious is identifying ME as the owner of the current mental holdings. (Damasio, 2021, p. 157-158).

In his previous writings, Damasio differentiated between *core* consciousness and *extended* consciousness in terms of their different “scope”. Core consciousness was defined as a “minimal-scope kind” of consciousness, focused on “the here and now, unencumbered by much past and by little or no future” (Damasio, 2012, p. 168), in contrast to extended consciousness, which he characterized as a “big-scope kind” of consciousness, where “a substantial part of one’s life comes into play and both the lived past and the anticipated future dominate the proceedings” (p. 169). In the present book, however, Damasio departs from this conceptualization by arguing that what is “extended” is not consciousness but the *mind*:

The problem, as I see it today, is that I should have talked, all along, about Extended Mind rather than Extended Consciousness. The fundamental mechanism whereby images are rendered conscious remains the same when the device is applied to a million images or to only one. (Damasio, 2021, p. 146)

The fundamental mechanism of consciousness, as Damasio sees it, does not rely on language, reasoning, future-orientation, autobiographical memory, or other typical human capacities, but is present already in non-human animals.

What then is this “fundamental mechanism”, whereby living organisms come to identify their experiences as theirs? In other words: How to explain “ownership knowledge”? I can’t see that Damasio provides any clear and unambiguous answer to this question, although he makes several interesting points on this topic. In particular, I find his idea about consciousness as “a state of mind imbued with two striking and related features: the mental contents it displays are *felt*, and those mental contents adopt one singular *perspective* (Damasio, 2021, p. 120) to be highly interesting.

Could it be that consciousness emerges as an amalgam of *feelings* and *perspective* (in rough analogy with how feelings may possibly have emerged as an amalgam of neuronal and biochemical processes, according to the discussion above)? In the following discussion I will refer to this possible hypothesis as *the feelings+perspective formula*.

In his discussion of our experience of “owning” our mind and body, Damasio attributes an important role to the individual’s homeostatic feelings:

Ownership knowledge can be obtained from specific facts and, quite directly, from homeostatic feelings. Easily, naturally, and instantaneously, as often as needed, homeostatic feelings *identify* my mind with my body, unequivocally, no extra reasoning or calculation needed. (Damasio, 2021, p. 158).

But are homeostatic feelings *sufficient* to identify the body as *my* body?

In phenomenologically oriented research, it is common to differentiate between the experience of *having* a body and the experience of *being* this body (e.g., Lundh & Foster, 2024). The experience of *being* a body involves the experience of *feeling the body “from within”,* whereas the experience of *having* a body involves the body as an *object* that can be seen (e.g., in the mirror), heard, touched, thought about, and evaluated. In other words, the latter involves the *perspective* that one has on one’s own body as an object among other objects in the physical world.

This suggests the possibility that the experience of *my body* relies not only on homeostatic bodily feelings but also on the ability to establish a *perspective* on one’s body an “object” in the world (cf. the notion of experienced embodiment as a passive synthesis of having a body and being this body; Lundh & Foster, 2024). This seems clearly compatible with the *feelings+perspectives formula*, although I can’t see that Damasio applies this reasoning explicitly to our conscious experience *of the body*. When he speaks about perspective it seems to be about the images we have of the external world, not of the body.

The *feelings+perspective formula* might well be applicable to all forms of consciousness, even though I have not seen Damasio develop this theme in full. For example, it might be applied also to our consciousness of *feelings* – which might possibly solve some apparent contradictions in Damasio’s reasoning about the relationship between consciousness and feelings.

For example, I felt a bit confused when reading Damasio’s (2021) claim that “*all* feelings are conscious” (p. 82). How is this compatible with his three-stage developmental model, where he seems to place the emergence of feelings at the second stage, whereas the development of the conscious mind belongs to the third stage? Moreover, in previous writings he has presented what I felt to be a rather convincing argument why all feelings are *not* conscious:

we *tend* to be conscious of our feelings. There is, however, no evidence that we are conscious of *all* our feelings, and much to suggest that we are not. For example, we often realize quite suddenly, in a given situation, that we feel anxious or uncomfortable, pleased or relaxed, and it is apparent that the particular state of feeling we know then has not begun on the moment of knowing but rather sometime before. (Damasio, 2000, p. 36)

To me, this implies that we have feelings whether we pay attention to them or not, and that feelings exist even before we become aware of them. In that same work, Damasio also seems to suggest a “three-step process” when it comes to the awareness of feelings:

I am suggesting that “having a feeling” is not the same as “knowing a feeling”, that reflection on feeling is yet another step up. (Damasio, 2000, p. 284)

Maybe a solution to this problem would be to apply the *feelings+perspective formula* also to *feelings*? This would mean that the consciousness of feelings would have two aspects: (1) the *feeling* of our feelings, and (2) the *perspective* on our feelings, as “objects” to reflect upon. Although it may seem somewhat awkward to speak about “feeling feelings”, Damasio used this verbal construction in a chapter named “Feeling feelings” in one of his previous books (Damasio, 2000, p. 279-295). Here he emphasizes that *feeling* a feeling is something more than *having* a feeling – and that reflection on one’s feelings is “yet another step up”.

This is a view on feelings which is compatible with how feelings are talked about in psychotherapy. There are many different schools of psychotherapy, but in one way or another they all attach importance to being conscious of one’s feelings (as well as of other experiences, including thoughts and images). In some forms of treatment, the focus is on the *feeling* component, as illustrated in formulations such as “stay with your feelings” and about the importance of “exposing” oneself to one’s anxieties. In other forms of treatment, the focus is more on the *perspective* component, as illustrated in the emphasis on the importance of reflecting on one’s experiences to arrive at emotional insights, or on the importance of reevaluating the “automatic thoughts” that are supposed to engender one’s feelings.

All this points to consciousness as a fundamental mechanism that can be applied to all sorts of “objects”, and that can expand our mind in all kinds of directions. For example, a focus on expanding *perspectives*, but with important feelings attached to both the process and the outcome, is found in science and philosophy. On the other hand, the arts and music are perhaps characterized primarily by their focus on *feelings,* although with important new perspectives attached.

On a more personal plane, the same duality between feelings and perspective can be seen in the development of a person’s autobiographic memory, and in their self-identity and experience of *personhood*:

Once experiences begin to be committed to memory, feeling and conscious organisms are capable of maintaining a more or less exhaustive history of their lives, a history of their interactions with others and their interaction with the environment, in brief, a history of each individual life as lived inside each individual organism, nothing less than the armature of personhood. (Damasio, 2021, p. 30)

Autobiographic memory, of course, involves both perspectives and feelings. Although the focus in memory research is mostly on the cognitive contents of memory, all kinds of memories also have emotional aspects. This is perhaps most obvious in the case of traumatic memories, but various researchers have also pointed to the positive feelings that may be attached to various memories – so-called “cherished memories”.

The expansive potentials of consciousness on the personal plane are clearly stated by Damasio:

Organisms endowed with conscious minds gain remarkable advantages. In keeping with their degree of intellect and creativity, their field of action widens. They can struggle for life in more varied settings. They can face a larger variety of hurdles and have a better chance of overcoming them. Consciousness expands their habitat. (Damasio, 2021, p. 133-134)

Damasio also makes clear that consciousness expands the individual’s *strivings* beyond homeostatic motives, thereby

making life not only possible but robustly so. The robustness of life is felt as “plenitude” and “flourishing” (Damasio, 2021, p. 96).

Conscious feelings in this way helps to pave the way for *flourishing*:

In the absence of feelings/consciousness, the mechanisms aligned with flourishing would not have gained favor so overwhelmingly. The presence of consciousness changed matters radically. (Damasio, 2021, p. 82).

Although most of what Damasio writes about consciousness points in the direction of consciousness as belonging to a later stage of evolution (the stage of *knowing*) than the stage where feelings enter the picture (the stage of *feeling*). But why then does Damasio in some passages state that “*all* feelings are conscious” (on p. 82)? I guess this is an expression of the *fluidity* of our present concept of “consciousness”. We simply have in mind several different kinds of phenomena which we want a word for, and “consciousness” seems to be the best word available in each of these cases.

## Conclusion

This is a book at the very frontiers of our growing understanding of sensing, feeling, and consciousness. There are many innovative parts of it, which may stimulate new thinking and new research on these matters. In particular, I found two themes to be of great interest: (1) Damasio’s views on feelings as hybrids between mental and physical processes that involve a two-way interaction between the interoceptive nervous system and the interior of our body; and (2) his conceptualization of the conscious experience as based on feelings and perspective.

Still, in thinking about these matters, I often feel that our existing terminology is insufficient. Discussions about the nature of sentience, feeling and consciousness all too often suffer from an insufficient differentiation between distinct kinds of phenomena. Some readers will probably object to Damasio’s reasoning about consciousness in this book because they mean something else by the word “consciousness”. Those who want to see consciousness as something much more *mysterious*, and who therefore speak about “the hard problem of consciousness”, will probably not be convinced by his reasoning.

An interesting perspective on the terminological issues is given by Damasio when he points out that the word “consciousness” is a relative newcomer in our language:

Unannounced and unaccompanied by a proper definition, the word “consciousness” has acquired multiple meanings and become a bit of a linguistic nightmare. The young English word did not even exist in the time of Shakespeare and has no direct counterpart in Romance languages; in French, Italian, Portuguese, and Spanish, one has to make do with the equivalent of “conscience” and use context to clarify which meaning of “conscience” the speaker is after. (Damasio, 2021, p. 119).

Languages develop and grow, and the terminology surrounding the phenomena which we today refer to as “consciousness” will probably be further differentiated by means of refined conceptualizations in the future – new conceptualizations that may also open up new perspectives and afford us new feelings of interest, excitement, and wonder (as yet another illustration of the fundamental mechanism of consciousness at work).


*Lars-Gunnar Lundh*


## Open access

This article is distributed under the terms of the Creative Commons Attribution 4.0 International License (http://creativecommons.org/licenses/by/4.0/), which permits unrestricted use, distribution, and reproduction in any medium, provided you give appropriate credit to the original author(s) and the source, provide a link to the Creative Commons license, and indicate if changes were made.
